# Expanding the Scope of an Amphoteric Condensed Tannin, Tanfloc, for Antibacterial Coatings

**DOI:** 10.3390/jfb14110554

**Published:** 2023-11-18

**Authors:** Somayeh Baghersad, Liszt Y. C. Madruga, Alessandro F. Martins, Ketul C. Popat, Matt J. Kipper

**Affiliations:** 1 School of Biomedical Engineering, Colorado State University, Fort Collins, CO 80526, USA; somayeh.baghersad@colostate.edu; 2 Department of Chemical and Biological Engineering, Colorado State University, Fort Collins, CO 80526, USA; liszt.coutinho_madruga@colostate.edu (L.Y.C.M.); alessandro.martins@uwrf.edu (A.F.M.); 3 Department of Chemistry & Biotechnology, University of Wisconsin-River Falls, River Falls, WI 54022, USA; 4 Department of Mechanical Engineering, Colorado State University, Fort Collins, CO 80526, USA; 5 School of Materials Science and Engineering, Colorado State University, Fort Collins, CO 80526, USA

**Keywords:** polyelectrolyte multilayer, Tanfloc, antibacterial activity, glycosaminoglycans, *N*,*N*,*N*-trimethyl chitosan, polyethyleneimine

## Abstract

Bacterial infections are a common mode of failure for medical implants. This study aims to develop antibacterial polyelectrolyte multilayer (PEM) coatings that contain a plant-derived condensed tannin polymer (Tanfloc, TAN) with inherent antimicrobial activity. Tanfloc is amphoteric, and herein we show that it can be used as either a polyanion or a polycation in PEMs, thereby expanding the possibility of its use in PEM coatings. PEMs are ordinarily formed using a polycation and a polyanion, in which the functional (ionic) groups of the two polymers are complexed to each other. However, using the amphoteric polymer Tanfloc with weakly basic amine and weakly acidic catechol and pyrogallol groups enables PEM formation using only one or the other of its functional groups, leaving the other functional group available to impart antibacterial activity. This work demonstrates Tanfloc-containing PEMs using multiple counter-polyelectrolytes including three polyanionic glycosaminoglycans of varying charge density, and the polycations *N*,*N*,*N*-trimethyl chitosan and polyethyleneimine. The layer-by-layer (LbL) assembly of PEMs was monitored using in situ Fourier-transform surface plasmon resonance (FT-SPR), confirming a stable LbL assembly. X-ray photoelectron spectroscopy (XPS) was used to evaluate surface chemistry, and atomic force microscopy (AFM) was used to determine the surface roughness. The LDH release levels from cells cultured on the Tanfloc-containing PEMs were not statistically different from those on the negative control (p > 0.05), confirming their non-cytotoxicity, while exhibiting remarkable antiadhesive and bactericidal properties against *Pseudomonas aeruginosa* (*P. aeruginosa*) and *Staphylococcus aureus* (*S. aureus*), respectively. The antibacterial effects were attributed to electrostatic interactions and Tanfloc’s polyphenolic nature. This work underscores the potential of Tanfloc as a versatile biomaterial for combating infections on surfaces.

## 1. Introduction

Implant-associated infections can cause serious complications, including tissue loss and sepsis. Millions of patients suffer from tissue loss due to infections on medical implant surfaces [1]. Tissue-engineered biomaterials provide mechanical, biological, and chemical support for cells to improve tissue healing around implants. However, microbial colonization not only inhibits healing, but also may lead to biofilm formation and sepsis. Some infections resist antibiotic treatment, particularly when biofilms are formed [2]. Predicting and preventing these infections is difficult due to the complex mechanisms of microbial adhesion, varying among different pathogenic bacterial strains, and due to the rise of multi-drug resistant bacteria. Introducing antibacterial activity to scaffold or medical implant surfaces is crucial for mitigating implant- and scaffold-associated infections. Antimicrobial surfaces should be developed with efficacy against various pathogens, including Gram-positive *S. aureus* and Gram-negative *P. aeruginosa* [3,4]. Approaches such as modifying surface chemistry, wettability, and topography have been investigated to prevent bacterial infections on biomaterial surfaces [5,6]. Alternative antimicrobial agents such as polymers, peptides, metals, and carbon nanomaterials are also receiving attention [7,8]. Modifying scaffold and implant surfaces to prevent bacterial adhesion and eradicate bacteria through multiple mechanisms may be an effective strategy to combat biofilm formation [9,10].

Polyelectrolyte multilayers (PEMs) have emerged as a versatile approach for surface modification, particularly in the design of antibacterial coatings [11,12]. The layer-by-layer (LbL) assembly process used to create PEMs offers precise control over thickness, composition, and surface chemistry. Moreover, they can be applied to a wide variety of substrates, including complex shapes like stents and implantable medical devices [13,14,15,16]. The versatility of PEMs allows for incorporating a wide range of polymers, nanoparticles, proteins, drugs, and cells, making them highly customizable for specific applications. Biopolymers are well-suited for PEM coatings in biomedical applications due to their biocompatibility, biodegradability, and similarity to biomolecules in the human body [17,18]. Through control over composition, surface charge, and degradation, PEMs can be designed to selectively control cell attachment, exhibit antibacterial properties, or release bioactive compounds in a controlled manner [19,20]. PEMs can be tailored to exhibit adhesion-resistant, contact-killing, or antimicrobial-agent-leaching characteristics, offering multiple strategies to limiting microbial colonization on material surfaces [11,13]. Therefore, the LbL deposition of PEMs provides a promising approach for designing and fabricating antibacterial coatings with tailored properties for various applications [21,22].

Tanfloc (TAN) is a tannin-based product derived from the *Acacia mearnsii* (black wattle) tree [23,24]. It is an amino-functionalized polyphenolic condensed tannin derivative that our research group has recently promoted for use in biomaterials. TAN has demonstrated broad-spectrum antimicrobial activity, attributed to its antiadhesive properties, high hydrophilicity, and interaction with bacterial cell walls and phospholipids through its amine groups [25,26]. Our studies have explored blending TAN with different polymers to enhance bacterial growth inhibition. The resulting TAN-based materials have exhibited improved antibacterial effects, biocompatibility, minimal toxicity toward mammalian cells, and biodegradability. The amine functional group makes TAN behave as a weak polycation in acidic solutions. TAN’s antimicrobial activity and polycationic nature make it an attractive polycation for PEMs designed to resist bacterial adhesion [27,28]. Rufato et al. [29] combined TAN with the biologically derived polyanionic polysaccharides pectin and *iota*-carrageenan in PEMs and demonstrated significant prevention of the attachment and proliferation of both *S. aureus* and *P. aeruginosa*. Compared to other biologically derived polycations such as chitosan, PEMs containing TAN as the polycation have shown superior antimicrobial properties, attributed to catechol and pyrogallol groups. The antimicrobial activity of TAN-based coatings has been attributed to various mechanisms, including enzyme inactivation and trace metal ion chelation [30]. These favorable properties make TAN a promising candidate for biomaterial applications.

Glycosaminoglycans (GAGs) are polyanionic polysaccharides that exhibit favorable characteristics for biomaterial applications, including non-toxicity, biodegradability, cytocompatibility, and the ability to enhance cell attachment and growth [31,32,33]. GAGs, such as heparin (HEP), hyaluronic acid (HA), and chondroitin sulfate (CS), can be used in nanomaterials to stabilize growth factors by preventing enzymatic degradation and facilitating growth factor–receptor binding [34,35]. Combining polyanionic GAGs with polycationic TAN can modulate the properties of biomaterials [6]. HEP, a highly negatively charged polymer, inhibits blood coagulation by preventing thrombin activation, making it a valuable component in blood-contacting biomaterials [36,37]. Combining the cationic tannin derivative TAN with HEP in PEMs results in a significant decrease in factor XII activation, platelet adhesion, and activation, enhancing blood compatibility and antibacterial properties on titanium surfaces [38]. 

While TAN has been successfully used as a polycation in PEMs, in this work, we hypothesize that the weakly acidic phenolic groups in TAN could also impart suitable polyanionic behavior at elevated pH to prepare PEMs containing TAN as the polyanion. We further hypothesize that the nature of the TAN complexation with the counter-polyelectrolyte (using TAN as either a polyanion or a polycation) will primarily complex either its amine groups or its catechol groups, leaving the other functional group available for imparting antimicrobial activity. By incorporating TAN as a polyanion in PEMs, we provide an innovative approach that expands the repertoire of TAN-based materials available for biomedical applications. Moreover, combining TAN with favorable polycationic polymers, such as *N*,*N*,*N*-trimethyl chitosan (TMC), supports PEMs with enhanced tissue compatibility and antibacterial activities. This further highlights the versatility and potential of TAN as a polyanion in the design of PEMs with enhanced properties for various biomedical applications [29]. This study describes the development of PEMs by assembling TAN (as a polycation) with HEP, CS, and HA, as well as TAN (as a polyanion) with TMC and polyethyleneimine (PEI) on oxidized glass surfaces. We demonstrate that these TAN-based biocompatible PEM assemblies have potent antiadhesive and antimicrobial properties against *P. aeruginosa* and *S. aureus*.

## 2. Materials and Methods

### 2.1. Materials

Tanfloc, an amino-functionalized polyphenolic tannin derivative with a molecular mass of approximately 600 kDa, was generously donated by Tanac SA (Montenegro-RS, Brazil) [39]; HEP, a sulfated glycosaminoglycan derived from porcine intestinal mucosa (12.5% sulfur, Mw = 14.7 kDa), was obtained from Celsus Laboratories (Cincinnati, OH, USA); HA salt derived from *Streptococcus equi* (Mw = 1.5 × 103 kDa) and CS salt sourced from shark cartilage (6% sulfur, 6-sulfate/4-sulfate = 1.24, Mw = 84.3 kDa) were purchased from Sigma-Aldrich (Saint Louis, MO, USA); PEI (Branched, Mw = 70 kDa) was obtained from Polyscience Inc. (Philadelphia, PA, USA); sodium acetate and sodium hydroxide were purchased from Fisher Scientific (Waltham, MA, USA); glacial acetic acid and ethanol (>98 vol%) were purchased from Acros Organics (Morris Plains, NJ, USA); 11-mercaptoundecanoic acid (MUA, 95%) was purchased from Sigma-Aldrich; TMC was synthesized by the single-step methylation of chitosan (deacetylation degree > 75% and Mw = 87 kDa) using methyl iodide as we have previously reported [40], and distilled water (DI water) with a resistivity of 18.2 MΩ cm obtained using a Millipore water-purification unit. The methylation of TMC was characterized by proton NMR in D_2_O (Supplementary material Figure S1). Additionally, membranes for dialysis with a molecular weight cutoff of 10 kDa were purchased from Sigma-Aldrich. Human adipose-derived stem cells (ADSC) were isolated by Prof. Kimberly Cox-York of the Department of Food Science and Human Nutrition (CSU) for a previous study from abdominal and femoral subcutaneous adipose tissue biopsy surfaces. The protocol for ADSC isolation from healthy individuals was approved by Colorado State University Institutional Review Board [41].

### 2.2. Tanfloc Purification

The commercial TAN product contains low-molecular weight material and hydrolyzable tannins that can be removed by dialysis. A solution of TAN in sodium acetate buffer (pH 5.0 and 0.2 mol L^−1^) was prepared with a concentration of 10 g/L and stirred overnight for complete dissolution. The TAN solution was then subjected to dialysis using a 10 kDa molecular weight cutoff dialysis tube for 72 h. Dialysis removes the chloride ions in excess in the TAN structure provided from the ammonium chloride used in the TAN synthesis [42]. The dialysate (DI water) was changed twice daily to ensure purification during dialysis. After the three-day dialysis, the solution was filtered using a Whatman filter paper (110 mm) to remove any remaining impurities. Subsequently, the filtered TAN solution was frozen at −80 °C and subjected to lyophilization for three days to obtain a dry, purified TAN.

### 2.3. Tanfloc Solubility by Dynamic Light Scattering (DLS)

The hydrodynamic diameter of TAN solutions was measured using a Zetasizer Nano ZS instrument from Malvern (Worcestershire, UK). Solutions were prepared by dissolving 2 mg/mL of TAN in solvents with different pH values (5, 6, 7.4, 8.4, and 9.3). The solutions were stirred overnight and then filtered using 0.22 μm syringe filters. DLS measurements were conducted immediately after sample preparation. Measurements were performed at 25 °C with a fixed scattering angle of 173° to determine the hydrodynamic diameter of the dissolved TAN particles. Each sample was measured three times, and the sample preparation process was repeated two times. The obtained hydrodynamic diameter and polydispersity index (PDI) values are reported as the average ± standard deviation.

### 2.4. Polyelectrolyte Multilayer Preparation

**Figure 1:**
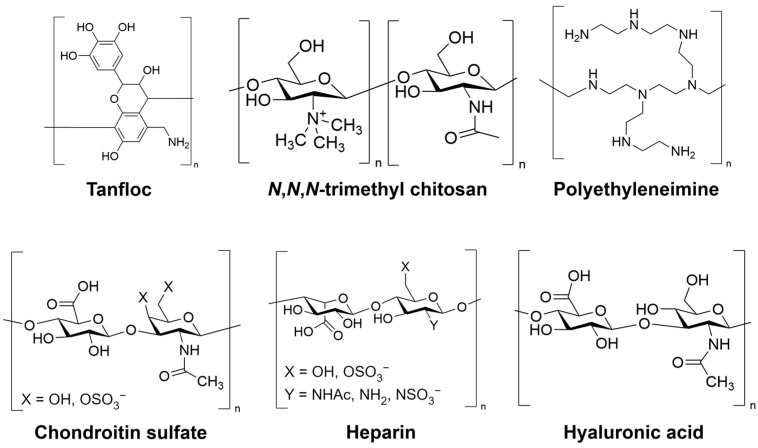
Chemical structure of polymers used in PEM preparation.

The experimental procedure used to prepare the PEMs is adapted from methods reported before by our research group [38,39]. HEP, HA, CS, PEI, and TMC solutions at a concentration of 1.0 mg mL^−1^, as well as TAN solution at a concentration of 2.0 mg mL^−1^, were prepared in a 0.2 M sodium acetate buffer solution with a pH of 5.0. The solutions underwent overnight stirring, and the pH of TAN (used as the polyanion), PEI, and TMC solutions was adjusted to 8.4 by gradually adding aqueous 1 M NaOH. For the rinse solutions, acidic and alkaline solutions were prepared using aqueous acetic acid (pH 4.0) and aqueous NaOH (pH 9.0), respectively. All solutions were filtered using 0.22 μm polyvinylidene fluoride (PVDF) syringe filters from Fisher Scientific (Waltham, MA, USA). The LbL deposition was performed on oxidized glass surfaces with a diameter of 12 mm and a thickness of 0.15 mm. The glass surfaces were placed in 24-well plates and were modified via oxidation using oxygen plasma to facilitate the deposition of the polycation. The rinse and deposition steps were all conducted on an orbital shaker (100 rpm). Before PEM deposition, the oxidized glass surfaces were rinsed by adding the rinse solution for 4 min. The rinse solution was then aspirated, and the polycation solution was added to the oxidized glass surface. After a 5 min adsorption, the polycation solution was removed, and the surface was rinsed for 4 min. Subsequently, the rinse solution was aspirated and the polyanion solution was deposited onto the oxidized glass surface, which already contained one layer of polycation. This deposition process was repeated to achieve either a 12-layer PEM terminated with a polyanion, or a 13-layer PEM terminated with a polycation. The PEM samples used in this study were labeled based on the polycation–polyanion pair and the layer number, such as TAN-HEP_12_. The chemical structures of the polyelectrolytes are shown in Figure 1.

### 2.5. Characterization

In situ Fourier-transform surface plasmon resonance: The method of PEM deposition using FT-SPR was adopted from previous studies [42]. Gold-coated glass chips with a 47 nm gold thickness were modified with a self-assembled monolayer of 1 mM MUA solution in ethanol for 24 h. The LbL assembly was conducted in the flow cell of an SPR-100 module coupled to a Nicolet 8700 FT-IR spectrometer (Thermo Fisher Scientific, Waltham, MA). FT-SPR measurements were performed with a white light/near-infrared source, a CaF_2_ beam splitter, and an InGaS detector. Data collection spanned the range from 6000 cm^−1^ to 12,000 cm^−1^ at 8 cm^−1^ resolution using Omnic 7.3 software (Thermo Fisher Scientific, Waltham MA). The intensity as a function of wavenumber for *p*-polarized light reflected from the back side of the gold film was measured to monitor the LbL assembly of the PEM in real-time. Solutions of polycation, polyanion, and rinse were flowed through the cell at a rate of 1 mL/min using a peristaltic pump. The PEMs were assembled by flowing the solutions in the following sequence: polycation, rinse, polyanion, and rinse. Each solution flowed for 5 min, and this process was repeated until 12 or 13 layers were deposited.

X-ray photoelectron spectroscopy: The composition of the PEMs was analyzed using XPS (5800 spectrometer, Physical Electronics, Chanhassen, MN, USA). A survey scan was conducted over the energy range of 10 to 1100 eV, with a pass energy of 187 eV. High-resolution spectra were obtained specifically for the carbon (C1s) and nitrogen (N1s) envelopes, using a pass energy of 23 eV. The acquired spectra were then subjected to spectral analysis using MultiPak software (Physical Electronics, Chanhassen, MN, USA) for peak fitting and composition characterization.

Atomic force microscopy: Surface morphology and roughness of the PEMs in phosphate-buffered saline (PBS pH 7.4) were evaluated using a BioScope Resolve BIOAFM (Bruker, Billerica, MA, USA) with Nanoscope V controller. The ScanAsyst mode was employed, utilizing a V-shaped silicon nitride cantilever on a pre-calibrated PFQNM-LC probe (Bruker, Billerica, MA, USA) with a spring constant of ~0.07 N/m. During topographical imaging, a peak force setpoint of approximately 1 nN was selected, optimized using the Bruker NanoScope software (Bruker, Billerica, MA, USA). All imaging was conducted at room temperature, and representative images were captured from at least two non-overlapping areas. The acquired images were analyzed using NanoScope Analysis version 2.0 software (Bruker, Billerica, MA, USA). The scan size was set at 2 × 2 μm^2^, with a digital resolution of 256 px × 256 px. The root-mean-square surface roughness (*R_q_*) was determined using Equation (1).

(1) $$R_{q}=\sqrt{\frac{\sum_{i}{\left(z_{i}-\bar{z}\right)}^{2}}{N}}$$

where $z_{i}$ is the distance of the *i*-th pixel from the mean height, $\bar{z}$, and *N* is the number of pixels.

### 2.6. Cytocompatibility Assay

The toxicity of the PEMs towards ADSC cells was evaluated using the lactate dehydrogenase (LDH) cytotoxicity assay [43]. ADSC cells at passage 6 were cultivated in Dulbecco’s Modified Eagle Medium (DMEM) supplemented with 10% fetal bovine serum and 1.0% penicillin/streptomycin and maintained at 37 °C and 5% CO_2_. Before cell seeding, the samples were sterilized with 70% ethanol for 30 min (*n* = 4). For the LDH assay, ADSC cells were seeded directly onto PEM and control surfaces at 20,000 cells/mL concentration in 24-well plates. Cells on polystyrene (PS) served as the negative control for cytotoxicity (C−), and cells on polystyrene treated with Triton X in the media (1.0 vol%) served as the positive control (C+) for cytotoxicity. After 24 h of incubation, the culture media from each well was collected and added to an equal amount of LDH substrate reagent solution (Quantichrom Bioassay Systems, Hayward, CA, USA) in a 96-well plate. The mixture was incubated for 30 min. The absorbance of the solution in each well was measured at 490 nm and 680 nm using a plate reader (FLUOstar Omega, BMG LABTECH, Cary, NC, USA). 

### 2.7. Antibacterial Activity Studies

The previously established procedures [38] were followed for the antibacterial activity assay. In brief, the antibacterial activity of different PEMs was assessed against Gram-negative *P. aeruginosa* (ATCC 10145) and Gram-positive *S. aureus* (ATCC 6538). A nutrient broth media solution (NBM, tryptic soy broth, Sigma) was prepared at 30 g/L. The bacteria pellets were re-suspended in the broth media solution and incubated at 37 °C for 24 h. The bacteria solutions were diluted to a concentration of 10^6^ CFU/mL to determine the antibacterial activity. Subsequently, 500 μL of the bacterial solution was exposed to the surfaces for 6 h and 24 h. After the incubation period, a 200 μL aliquot of the solution was extracted, and the optical density at 560 nm was measured using a plate reader. Under identical conditions, control samples of glass and polystyrene (PS) were also included. The calculation of bacterial growth inhibition in solution was performed by comparing the obtained values to the control.

The quantification of live and dead bacteria adhering to the surfaces was performed using fluorescence microscopy (Zeiss Axiovision, Jena, Germany) at 20× magnification. Following the incubation for 6 h and 24 h, the bacteria solution was removed, and the surfaces were rinsed three times with PBS to eliminate non-adhered bacteria. Subsequently, the surfaces were incubated in a stain solution consisting of propidium iodide and SYTO 9 in PBS (3 μL/ml, 1:1). After a 15 min incubation period in a dark environment at room temperature, the stain solution was aspirated, and the surfaces were rinsed with PBS. Then, the cells were fixed with 3.7% formaldehyde for 15 min at room temperature and washed thrice in PBS. The surfaces were immediately visualized using a fluorescence microscope (*n* = 3). The percentages of live and dead bacteria on the surfaces were determined using ImageJ software (version 1.54g, NIH, Bethesda, MD, USA).

Bacteria morphology and biofilm formation on PEMs were analyzed using scanning electron microscopy (SEM, JEOL JSM-6500F, Peabody, MA, USA). Following the incubation period (6 h and 24 h), samples were fixed in the fixative solution (3% glutaraldehyde, 0.1 mol/L sodium cacodylate, and 0.1 mol/L sucrose) for 45 min at room temperature. Subsequently, the samples were washed with a buffer (0.1 mol/L sodium cacodylate and 0.1 mol/L sucrose) for 10 min. The samples were then subjected to drying with increasing concentrations of ethanol solutions (35, 50, 75, and 100%) for 10 min each. Before SEM imaging, the surfaces were coated with a thin layer of gold at a thickness of 10 nm (*n* = 2) and the SEM parameters were optimized and chosen as follows: accelerating voltage of 15 kV, working distance of 10 mm, and vacuum pressure below 3 × 10^−4^ Pa.

### 2.8. Statistical Analyses

At least three different samples of each sample were used in all experiments; results are presented as mean ± standard deviation. Differences were determined using one-way ANOVA (*p* = 0.05) with a post-hoc Tukey’s honest significant difference test.

## 3. Results and Discussion

### 3.1. DLS

This study evaluated the solubility of TAN, an amphoteric polymer with amine and pyrogallol/catechol groups imparting basicity and acidity, respectively, under different pH conditions using DLS measurements (Table 1). 

The results revealed notable variations in dissolved TAN’s hydrodynamic diameters and PDI at various pH values. Specifically, the smallest hydrodynamic diameters with the lowest PDI were observed under acidic conditions at pH 5.0, indicating a high degree of particle size uniformity and fully dissolved TAN polymer chains. This pH was chosen for preparing polycationic TAN because the presence of substituted amine groups, with a pK_a_ value of approximately 6.0 [39], results in positive charge density at pH values below 6.0. TAN undergoes charge loss when the pH exceeds the pK_a_ of its protonated amine groups. This leads to polymer chains interacting with each other instead of dissolving in the solvent, resulting in agglomeration and a significant increase in the hydrodynamic diameter. At pH 7.4, the hydrodynamic diameter is very low, indicating some collapsed polymer chains, but the PDI of 1.00 indicates high molecular heterogeneity. This also suggests that many particles were likely filtered out via a 0.22 µm filter, likely substantially reducing the polymer concentration.

TAN is amphoteric, containing both basic amine groups and acidic catechol/pyrogallol groups; the phenolic hydroxyl groups undergo deprotonation with a pK_a_ value falling within the range of 8.3 to 8.7, imparting negative charges [44]. Compared to pH 9.3, the deprotonation of hydroxyl groups at pH 8.4 is likely optimized, resulting in the formation of smaller and more soluble particles. Consequently, this optimization contributes to a lower PDI, indicating enhanced particle size and solubility uniformity. Additionally, a qualitative assessment of the solubility of the solutions was conducted based on their color. The solutions at various pH values showed a brownish color, indicating solubility, except for pH 7.4, where the solution was completely clear. This also suggests that poor solubility at pH 7.4 caused a substantial amount of material to be removed via the 0.22 μm pore size filtration step.

Previous studies have demonstrated that the solubility of TAN can be influenced by hydrolysis–hydration processes, particularly under highly alkaline conditions (pH > 10), which may compromise the stability and solubility of the polymer structure [39]. Hence, pH 5.0 and 8.4 were selected as representative conditions for the use of TAN as a polycation and polyanion, respectively. 

### 3.2. PEM Assembly and In Situ FT-SPR

In this paper, the results of TAN-HEP and TMC-TAN PEMs were chosen to be presented as representatives of PEMs that contain TAN as a polycation and polyanion, respectively. The characterizations of these PEMs are discussed in detail throughout the main body of the paper, while the results of other PEMs can be found in the supplementary information.

**Figure 2:**
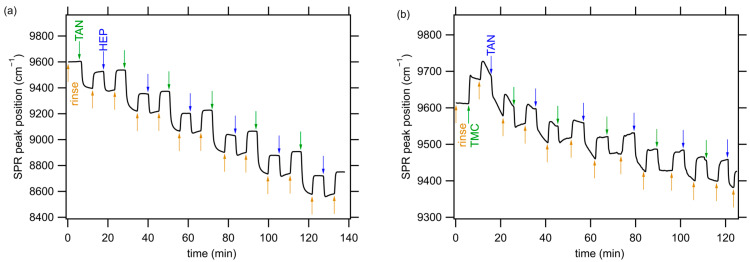
Kinetics of 12-layer PEM assembly of (**a**) TAN-HEP_12_ and (**b**) TMC-TAN_12_ monitored by in situ FT-SPR. Arrows indicate the start of rinsing (orange), polycation deposition (green), and polyanion deposition (blue) steps. Representative SPR data for PEMs containing TAN paired with HA, CS, and PEI are shown in the supporting information (Figure S2).

The LbL assembly of PEMs was monitored using in situ FT-SPR. A representative set of results obtained during the LbL assembly of TAN-HEP_12_ and TMC-TAN_12_ PEMs is presented in Figure 2. The supporting information shows the SPR data for the additional PEMs using TAN as a polycation (TAN-HA_12_, TAN-CS_12_) and TAN as a polyanion (PEI-TAN_12_). The experimental procedure for the TAN-HEP_12_ PEM began with a five-minute rinse step (the orange arrow in Figure 2a) on a gold-coated glass chip modified with MUA. Subsequently, the polycation solution was introduced into the flow cell, starting at the green arrow in Figure 2a. This caused the position of the SPR peak to shift to a lower wavenumber value due to the increase in the refractive index near the surface associated with TAN adsorption. After the initial adsorption of the polycation, the surface was rinsed for another five minutes, increasing the peak position of the SPR signal, though not to the value of the original rinse, because of the irreversibly adsorbed TAN. Next, the polyanion solution was introduced into the flow cell, indicated by the blue arrow in Figure 2a, and the process was repeated in a LbL approach until a 12-layer PEM was constructed. Upon adsorption of each successive bilayer, the PEM thickness increased, resulting in a shift in the SPR peak during rinse steps. The notable drop in the FT-SPR peak position was observed during each adsorption step and is attributed to two factors: the adsorption of the polyelectrolyte onto the surface and the refractive index difference between the rinse solution and the polymer solution. The difference in peak position before and after each adsorption step represents the irreversible adsorption of the charged polyelectrolyte.

The significant changes observed in the SPR absorption peak during PEM assembly confirm the LbL PEM assembly. Notably, TAN demonstrated the potential to function as both a negatively and positively charged component in PEM structures, with the adjustment of solution pH. Moreover, the thickness of the adsorbed PEMs depends on the specific polyanion–polycation pairs used and can vary due to the interactions between strong and weak polyelectrolyte combinations [45]. Previous investigations from our research group have demonstrated that polysaccharide-based PEMs formed under these conditions are remarkably thin, measuring only a few nanometers [42]. For the TAN-HEP PEM, the SPR peak position exhibits a relatively large change following TAN deposition (>300 cm^−1^, for layers following the first layer), compared to the change associated with HEP deposition (>100 cm^−1^). This discrepancy can be attributed to the differences in molecular weight and charge density of the polymers, which result in different adsorption profiles. TAN has a significantly higher molecular weight and likely adsorbs in a coiled conformation, whereas HEP has a much lower molecular weight and has higher charge density imparted by many sulfate groups; it adopts a more extended conformation in solution [46]. 

When PEMs are formed with TAN as the polyanion and TMC as the polycation, the SPR peak position exhibits an increase (rather than a decrease) upon the initial TMC adsorption step due to the difference in the refractive index between the initial rinse and the TMC solution (Figure 2b). Adding subsequent layers results in a decreasing SPR peak position with each bilayer. Also, when TAN is used as the polyanion, there is a tendency for the TAN adsorption steps and their subsequent rinsing to fail to equilibrate during the five-minute rinse and adsorption steps. This could prove that the TAN slowly rearranges on the surface to adopt an optimal configuration. Similar slower equilibration is also observed for the PEI-TAN PEM assembly (see Figure S2 in the supporting information). Pairing TAN with TMC led to smaller refractive index changes and thinner layers than PEMs formed by pairing TAN with the polyanions. Pairing TAN with PEI led to the largest refractive index and thickness changes.

### 3.3. X-ray Photoelectron Spectroscopy 

**Figure 3:**
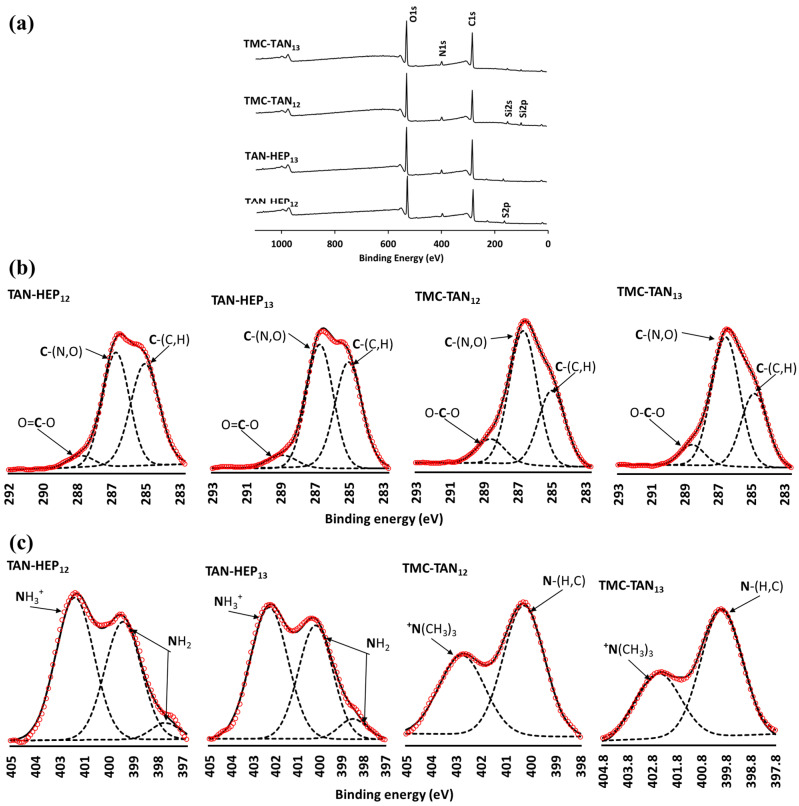
Survey (**a**) and high-resolution X-ray photoelectron spectra of the C1s (**b**) and N1s regions (**c**) of PEM surfaces. Red circles represent data; broken lines represent peak fits; solid line represents some of individual peak fits. (Survey scans of the PEI-TAN, TAN-CS, and TAN-HA multilayers are shown in Figure S3 in the supporting information).

The XPS analysis confirmed the surface compositions of all TAN-containing PEMs (Figure 3 and Figure S3 in the supporting information). Survey scans of TAN-HEP and TMC-TAN with different terminated layers are shown in Figure 3, and the survey scans of PEI-TAN, TAN-CS, and TAN-HA PEMs are shown in Figure S3 in the supporting information. The obtained XPS spectra revealed distinct characteristic peaks corresponding to carbon (C1s) at 284 eV, oxygen (O1s) at 530 eV, nitrogen (N1s) at 400 eV, sulfur (S2p) at 167 eV, and silicon (Si2p) at 102 eV (Figure 3a). The absence of a detectable silicon peak confirms the complete coating of the substrate using TAN-HEP PEMs. However, a slight detection of the Si peak in TMC-TAN PEMs indicates a thinner coating. As expected, all surfaces modified with PEMs exhibit N1s peaks, which are characteristic of the composition of the polyelectrolytes. The N1s peak can be attributed to TAN and TMC in the PEM coatings because TMC contains *N*-methylated and *N*-acetylated glucosamine residues, and TAN contains amine groups. The N1s peak intensity shows an increase in the PEMs terminated with these polymers, whereas in the PEMs terminated with HEP, there is a notably higher intensity in the sulfur (S2p) peak.

High-resolution XPS spectra for the C1s and N1s envelopes for TAN-HEP and TMC-TAN PEMs are shown in Figure 3b and Figure 3c, respectively. The high-resolution C1s spectra display three distinct sub-peaks, corresponding to aliphatic carbon, amine, carboxyl, and amide functional groups. The C1s envelopes provide signals at approximately 284–285 eV for C-C and C=C bonds and 287 eV for C-N bonds, which can be attributed to the phenolic and amine moieties present in the TAN chains. The peaks observed at 288.2 eV correspond to carboxylate (-COO^−^), carbonyl (C=O), and carboxylic acid (-COOH) groups, confirming the presence of HEP on the TAN-HEP surface.

Electrostatic interactions between cationic and anionic entities stabilize the PEMs. For the TAN-HEP PEMs formed at acidic pH, the polymer chains have sulfates and carboxylates (pKa 3.5) on HEP and protonated amines on TAN (pKa 6.0). For the TMC-TAN PEMs formed at pH 8.4, TMC exhibits permanent positive charges due to *N*-quaternization, while TAN experiences partial deprotonation of phenolic groups, resulting in the generation of negative charges. Based on the pH of assembly, the likely ion pairs in TAN-HEP PEMs formed at acidic pH are -NH_3_^+^ (on TAN) and SO_3_^−^ and COO^−^ (on HEP). The ion pairs in the TMC-TAN PEMs formed at alkaline pH are the -^+^N(CH_3_)_3_ (on TMC) and -CO^−^ (on TAN). Hydrogen bonding and ion-dipole interactions may also stabilize the PEMs.

These likely modes of ion pairing are supported by the high-resolution N1s spectra. The small peak at 397.8 eV arises from the amine pendent to the aromatic ring in TAN. The peak at 400 eV (-NH_2_ and N-C=O) arises from the amine and amide groups in the respective counter-polyelectrolytes [47]. The TAN-HEP PEM is assembled at acidic pH, in which the pendant amine on TAN is protonated and interacts electrostatically with negatively charged (carboxylate and sulfate) groups in HEP in these PEMs. In the TMC-TAN PEMs assembled at higher pH, this contribution is from methylated amine on TMC. While both TMC and TAN contain amine nitrogen that could be protonated, and in TMC, some of these groups are fully methylated to quaternary ammonium, the peak at 402.5 is weaker in the TMC-TAN PEMs than in the HEP-TAN PEMs. TMC-TAN PEMs are assembled at alkaline pH, preventing TAN amine protonation. This is further evidence that in this condition TAN interacts with TMC primarily through its deprotonated phenolic groups, rather than through its pendant amine. The ion-pairing differences determine which functional groups in TAN might be available for interacting with proteins, mammalian cells, and bacteria.

### 3.4. Atomic Force Microscopy 

**Figure 4:**
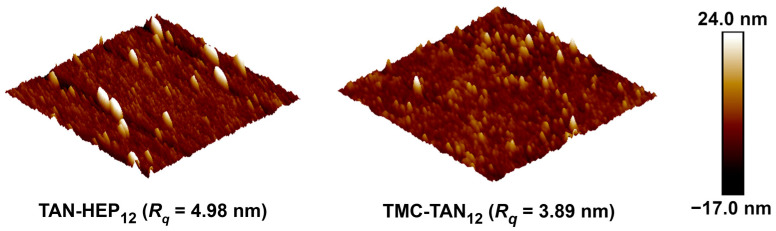
Representative 2.0 μm × 2.0 μm AFM topographic images of the PEMs taken in PBS. AFM images of TAN-HA, TAN-CS, and PEI-TAN PEMs are shown in the supporting information.

AFM analysis provides information on nanoscale and microscale surface characteristics, including three-dimensional topography and homogeneity, which influence surface biological activity. For this investigation, AFM was performed in a PBS environment to characterize the topological features of the PEMs on gold-coated chips. The AFM images in Figure 4 confirm the complete surface coverage, indicating the homogeneous deposition and assembly of the multilayered films. The surface roughness is characterized through the root-mean-squared roughness (*R_q_*) values. The surfaces exhibited similar surface roughness, though the TAN-HEP PEM has slightly larger features and a slightly rougher surface, perhaps due to stronger complexation between TAN and HEP to form polyelectrolyte complexes on the surface. AFM images of the TAN-HA, TAN-CS, and PEI-TAN PEMs are shown in Figure S4 in the supporting information.

Various factors, including the structure of ionic polymers, polymer concentration, molar mass, surface polarity, and PEM composition influence the surface topography of PEMs. Earlier studies have highlighted that weakly charged polyelectrolytes, like chitosan, tend to adopt a coiled conformation, leading to rougher surfaces [29]. Da Câmara et al. [46] reported that the TAN-CS PEM surface displayed relatively higher roughness than the TAN-HEP PEMs, as HEP contains more sulfated groups, contributing to its higher charge density.

In this study, the TAN-HEP PEM displayed a slightly higher roughness of 4.98 nm compared to TMC-TAN with a roughness of 3.89 nm. TAN is a weak polyelectrolyte with ionized moieties (-CO^−^ in alkaline pH and NH_3_^+^ in acidic pH) that primarily interacts with strong counter-polyelectrolytes (TMC and HEP) through coulombic and ion-dipole forces. Incorporating HEP, characterized by a molecular weight of 14.7 kDa, and utilizing linear structures like TMC tend to contribute to a lower surface roughness when combined with TAN. The TAN-HA PEM (supporting information Figure S4) comprises the two highest molecular weights and weakest polyelectrolytes, and has the highest surface roughness and largest-sized surface features. 

### 3.5. Cell Cytocompatibility 

To evaluate the applicability of the surfaces modified with TAN polyanion and polycation as tissue-contacting biomaterials, it is important to investigate the compatibility of the surfaces with primary human cells. The cytotoxicity of the surfaces was assessed using the LDH reaction method, which provides an estimate of the number of cells undergoing lysis or cell death in the culture media containing the polymers. LDH, an enzyme in the cytoplasm of cells, is released into the extracellular medium upon the loss of membrane integrity, which can occur due to apoptosis or necrosis [43,48]. Therefore, LDH is a reliable marker for evaluating cell membrane integrity and is useful for assessing the cytotoxicity of the PEMs. The released LDH enzyme can be quantified by reacting it with a tetrazolium salt, resulting in the formation of a red product that can be quantified by its absorbance at 490 nm.

**Figure 5:**
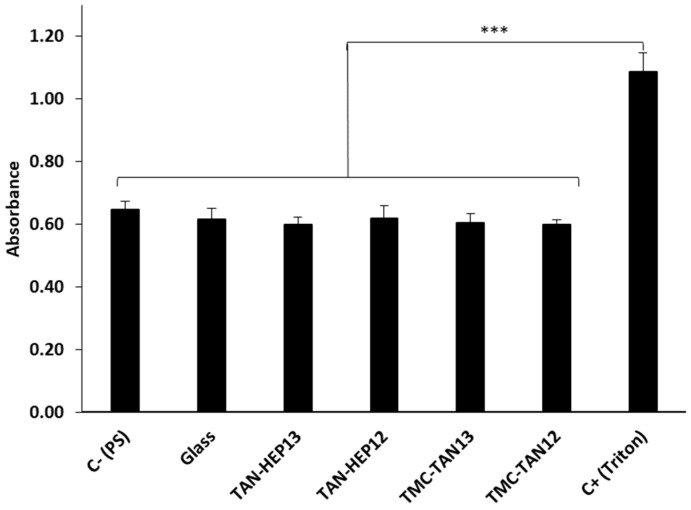
Cytotoxicity results assessed through LDH assay on different PEM surfaces after 24 h of incubation. Values represent mean ± standard deviation (*n* = 4). *** *p* ≤ 0.001 compared to the positive (Triton) control. Similar results for the TAN-HA, TAN-CS, and PEI-TAN PEMs are shown in Figure S5 in the supporting information.

Figure 5 presents the absorbance results from the LDH activity assay for ADSCs cultured for 24 h on the TAN-HEP and TMC-TAN PEMs with different terminated layers. Triton X, which completely lyses cells to release LDH, was used as the positive control. The negative control (PS) represents the spontaneous background release of LDH by healthy ADSCs cultured on tissue culture polystyrene. Notably, when the cells were incubated with the PEM surfaces, the measured LDH concentration was similar to the LDH level observed in the negative control (p > 0.05). These findings indicate that the tested samples did not exhibit cytotoxicity towards ADSCs. Similar results for the TAN-HA, TAN-CS, and PEI-TAN PEMs are shown in Figure S5 in the supporting information.

### 3.6. Antibacterial Activity Study

It is vital to develop biomaterial surfaces that can prevent bacterial adhesion, proliferation, and biofilm formation to address the issue of persistent biomaterial-associated infections. *P. aeruginosa* and *S. aureus* bacteria have gained significant attention due to their involvement in severe life-threatening infections. When bacteria colonize implant surfaces they form biofilms that protect them from conventional antibiotics. *S. aureus*, commonly found on human skin, is often associated with infections related to medical devices, while *P. aeruginosa* is frequently implicated in hospital-acquired infections and triggers oxidative stress and inflammation. Understanding how biomaterial surfaces interact with both types of bacteria is necessary, as they require different strategies for controlling their infections [49,50,51]. Glass and tissue culture polystyrene were chosen as negative controls because they do not have antimicrobial and antiadhesive activities [49].

*P. aeruginosa* and *S. aureus* were also chosen as model bacteria because they exhibit distinctive cellular envelope architectures. Gram-positive bacteria have a single lipid bilayer and a thick peptidoglycan cell wall. Gram-negative cells have an inner membrane, a thin layer of peptidoglycan, and an outer membrane. Glycoproteins are components of the S layer, the outermost layer of the cell envelope, while the cell wall consists mainly of peptidoglycan. The negative charge density observed in Gram-positive bacteria can be attributed to teichoic acid moieties, while Gram-negative bacteria derive their negatively charged walls from phospholipids [52,53]. These differences in cellular envelope structures and surface charge between the two bacterial types may influence their distinct interactions with the PEMs.

**Figure 6:**
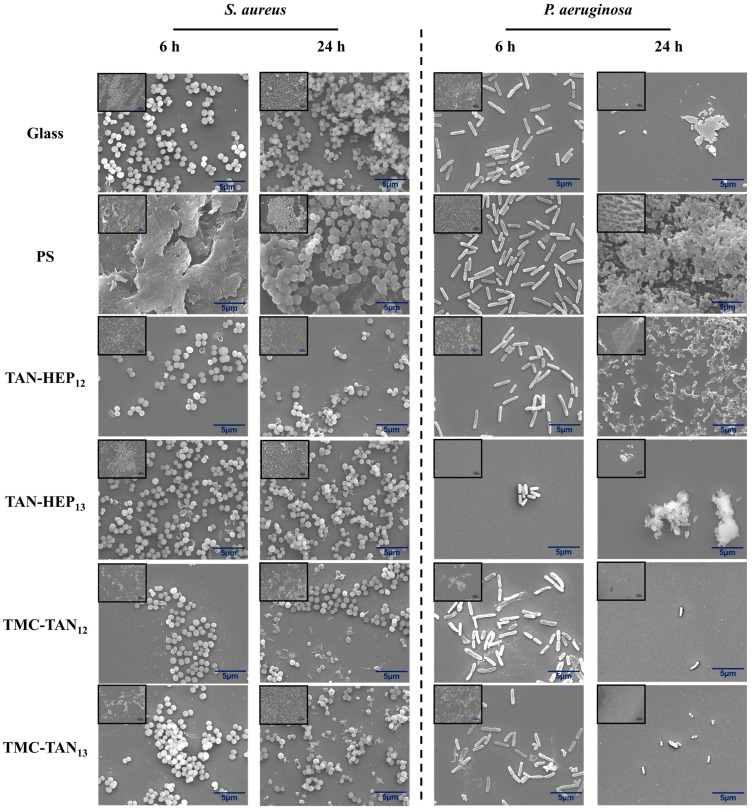
SEM images of the TAN-HEP and TMC-TAN PEMs with different terminated layers incubated with *S. aureus* and *P. aeruginosa* after 6 h and 24 h at 37 °C. Glass and PS were considered controls. Original magnification is 5000×, and 1000× (inset). SEM images of *S. aureus* and *P. aeruginosa* after 6 h and 24 h on TAN-HA, TAN-CS, and PEI-TAN surfaces are shown in Figure S7 in the supporting information.

SEM images (Figure 6 and Figure S7 in the supporting information) were utilized to assess the morphology of adhered bacteria and the formation of biofilms on the PEMs following incubation in a bacteria broth for 6 h and 24 h. After 6 h, fewer *S. aureus* bacteria with spherical morphology were observed on the PEM-coated surfaces compared to both control surfaces (glass and PS). Moreover, some bacteria on PEMs begin to exhibit morphological changes. After 24 h, the control surfaces displayed a high number of adhered bacteria accompanied by colony and biofilm formation. In contrast, the PEM surfaces exhibited a reduced number of adhered bacteria and smaller and fewer colonies. Also, some bacteria on the PEM-coated samples displayed defective membranes, suggesting their non-viability. Importantly, no biofilm formation was detected on any of the PEMs. It is important to note that after 6 h of incubation, our SEM analysis revealed a mature biofilm characterized by a dense extracellular polymeric substance matrix on PS surfaces, making individual bacterial cells indiscernible within the biofilm structure that shows these bacteria can easily form biofilm in favorable environments. This dense matrix is indicative of a well-established biofilm at this stage. However, at the 24 h time point, the SEM images clearly showed a distinct change. The biofilm structure appeared less compact, allowing individual bacterial cells to be observed. This observation strongly suggests the occurrence of biofilm dispersal.

It is crucial to highlight that *P. aeruginosa* is a biofilm-forming bacterium. This defense mechanism makes it a challenging pathogen to fight [53]. After 6 h, the control surfaces and PEMs, except those terminated with TAN, exhibited a substantial number of adhered *P. aeruginosa* bacteria, characterized by their bacillus morphology. Nevertheless, some disruptions in bacterial morphology were observed, indicating the presence of non-viable bacteria. After 24 h, PS still exhibited a higher number of adhered bacteria, along with colony and wide biofilm formation. In contrast, the PEMs displayed significantly fewer attached bacteria and no biofilm formation, indicating their significant antiadhesive activity (Figure 6 and Figure S7 in the supporting information). *S. aureus* with defective cell membranes are particularly evident in the PEMs in which TAN is used as the polyanion (TMC-TAN and PEI-TAN PEMs) after 24 h compared to the PEMs in which TAN is used as the polycation. This suggests that the availability of the amine in TAN, when it is used as a polyanion in PEMs may promote membrane disruption through interaction with teichoic acid moieties in the Gram-positive cell wall. In contrast, for the Gram-negative bacterium *P. aeruginosa*, membrane disruption and cell debris are most evident on the TAN-HEP and TAN-CS PEMs, in which TAN is incorporated as the polycation. This may be due to interactions of the free phenolic groups with lipoproteins and glycoproteins in the Gram-negative cell wall. Protein binding is a key feature of tannins imparted by their polyphenolic characteristics.

**Figure 7:**
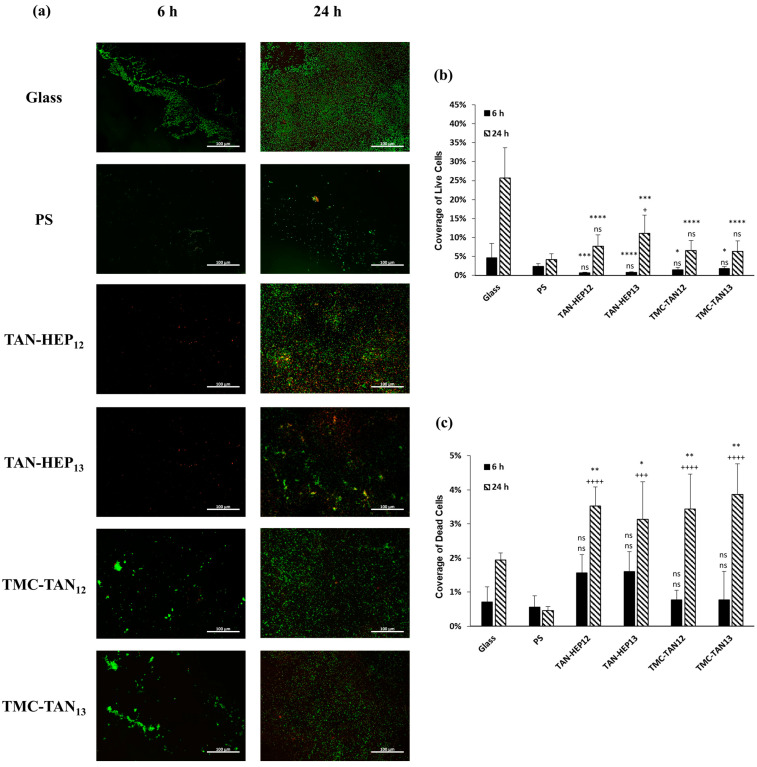
(**a**) Fluorescence microscopy images of *S. aureus* on the PEMs. Live bacteria are represented in green (SYTO 9 stain) and dead bacteria in red (propidium iodide stain). Percentage of coverage for (**b**) live and (**c**) dead *S. aureus* adhered to the surfaces. Glass and PS were considered as controls. **** *p* ≤ 0.0001, *** *p* ≤ 0.001, ** *p* ≤ 0.01, * *p* ≤ 0.05, and “ns” *p* ≥ 0.05 compared to glass control at same time point; ++++ *p* ≤ 0.0001, +++ *p* ≤ 0.001, + *p* ≤ 0.05, and “ns” *p* ≥ 0.05 compared to PS control at same time point. Fluorescence microscopy images of *S. aureus* on the TAN-HA, TAN-CS, and PEI-TAN PEMs are shown in Figure S8 in the supporting information.

**Figure 8:**
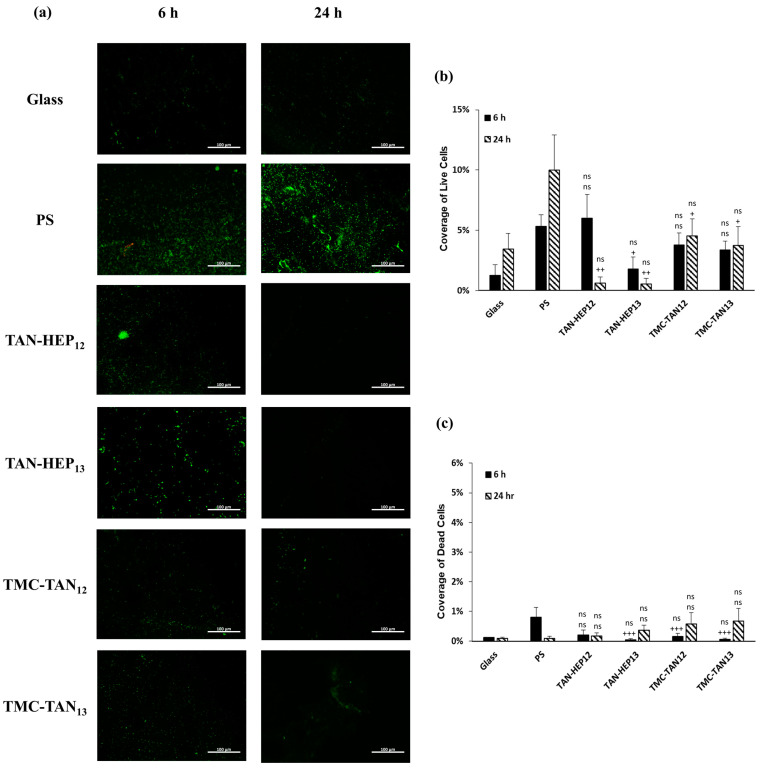
(**a**) Fluorescence microscopy images of *P. aeruginosa* on the PEMs. Live bacteria are represented in green (SYTO 9 stain) and dead bacteria in red (propidium iodide stain). Percentage of coverage for (**b**) live and (**c**) dead *P. aeruginosa* adhered to the surfaces. Glass and PS were considered as controls. “ns” *p* ≥ 0.05 compared to glass control at same time point; +++ *p* ≤ 0.001, ++ *p* ≤ 0.01, + *p* ≤ 0.05, and “ns” *p* ≥ 0.05 compared to PS control at same time point. Fluorescence microscopy images of *P. aeruginosa* on the TAN-HA, TAN-CS, and PEI-TAN PEMs are shown in Figure S8 in the supporting information.

Figure 7, Figure 8 and Figure S8 in the supporting information present fluorescence microscopy images and the corresponding percentage coverage of live and dead *S. aureus* and *P. aeruginosa* bacteria on the surfaces coated with PEMs at 6 h and 24 h, respectively. In general, there was a higher adhesion of *S. aureus* bacteria observed on all PEMs compared to *P. aeruginosa*. Despite this higher adhesion, a significant number of the *S. aureus* bacteria attached to the PEM-coated surfaces were stained red, indicating that they were dead after 24 h of growth (Figure 7a). The PEMs had significantly more dead *S. aureus* after 24 h than either of the controls and significantly fewer live *S. aureus* after 24 h than the glass control. Notably, the biofilm observed in the SEM image in Figure 6 for the *S. aureus* on PS after 6 h may have prevented the cells in the biofilm from taking up the live and dead cell stains (Figure 7a). Therefore, it is important to interpret the fluorescence microscopy based on what is also observed in the SEM. No significant difference was observed among the PEMs in terms of their composition and terminated layer regarding the percentage of live and dead *S. aureus* bacteria (Figure 7b,c).

All TAN-based PEMs demonstrated a significant decrease in the adhesion of live *P. aeruginosa* bacteria compared to the control group after 24 h (Figure 8a). TAN-HEP PEMs exhibited a significantly greater reduction in bacterial adhesion than the control group and TMC-TAN PEMs. After 24 h, the bacteria attached to the TMC-TAN surface were mostly found to be non-viable, whereas the TAN-HEP surface showed almost no attached bacteria (Figure 8b,c). No significant difference was observed in the percentage area of live and dead bacteria between PEMs with different terminated layers. The quantitative results from the cell staining in Figure 7 should be interpreted in light of the significant biofilm formation of *P. aeruginosa* on the control surfaces shown in the SEM images in Figure 6. This biofilm formation may inhibit the transport and uptake of the stain molecules on the control surfaces. The primary antibacterial mechanism of these PEMs against *P. aeruginosa* is their antiadhesive characteristic, which inhibits bacterial adhesion to the surfaces. This is supported by both the SEM images (Figure 6), as the absence of biofilm formation on the PEM-coated surfaces suggests that they do not provide a suitable environment for bacterial adherence and growth, in contrast to the significant biofilm formation observed on the PS surfaces (Figure 6). The PEMs primarily inhibit bacterial colonization and growth on contact, without having a strong antimicrobial effect on bacteria in solution. (See Figure S6 in the supporting information). This suggests that the mode of action is not due to the leaching of components into the solution.

The presence of polyphenolic and cationic moieties in TAN-based PEMs contributes to their significant antibacterial properties against both *S. aureus* and *P. aeruginosa*, and the mechanism may depend upon whether TAN is incorporated as a polyanion or a polycation [54]. The amine groups in TAN can interact with the negatively charged bacterial cell wall. These interactions increase membrane permeability, resulting in the leakage of cellular constituents and ultimately cell death [55]. Additionally, the flavonoid-based structure of TAN contributes to its antimicrobial activity. Flavonoids have been shown to disrupt microbial membranes, form complexes with bacterial extracellular matrices, and promote bacterial cell death [56,57].

Some of the observed antimicrobial activity is also undoubtedly imparted by the other components of these PEMs. These combined effects are not decoupled in this study. The antibacterial activity of TMC primarily relies on its polycationic *N*-quaternized groups, which remain positively charged across a wide pH range and strongly interact with microorganism cell membranes, leading to effective antimicrobial inhibition under physiological pH conditions [58,59]. The flexible chains of TMC facilitate easier interactions with the bacterial cell wall. TMC also contains some amine, *N*-methyl, and *N*,*N*-dimethyl ammonium groups that contribute to its antibacterial activity through chelation effects, which inhibit microbial growth by binding to metallic cations present in cell walls, as well as hydrophobic effects in addition to electrostatic forces. The hydrophobic methyl groups in the *N*-quaternized groups further enhance the interaction with the lipid cell membrane, promoting improved antimicrobial activity [29,60].

Furthermore, studies demonstrate the potential antiadhesive capacity of polyanions. Specifically, the presence of ionized phenol (O^−^), sulfate (OSO_3_^−^), and carboxylate (COO^−^) groups on their surfaces imparts a negative charge density [42]. This negative charge density plays a crucial role in promoting the repulsion between the anionic phospholipid and the negatively charged polymer, effectively suppressing bacterial adhesion. Previous studies have also attributed the antiadhesive properties of heparin/chitosan-based PEMs to the negative charge density on the PEM surfaces [49].

This study reveals that TAN is a versatile component of PEM coatings for importing antimicrobial activity because of its amphiphilic character. When TAN is used as a polycation in the PEM, its amine groups are bound to the counter polyanion, and the catechol groups are available to interact, resulting in the substantial disruption of Gram-negative bacterial cell integrity. However, when TAN is used as a polyanion, its pyrogallol/catechol groups bind to the counter polycation, and the pendant amine disrupts Gram-positive cell membranes. Both types of PEMs resist the biofilm formation of *P. aeruginosa* and result in the substantial killing of *S. aureus* by contact.

## 4. Conclusions

While TAN has previously been used as a polycation in PEMs, this study demonstrates for the first time that the amphiphilic nature of TAN enables its use as either a polycation or a polyanion in biopolymer-based PEMs. This versatility enables a wide range of PEM compositions by selecting either a polyanion or a polycation as the counter polyelectrolyte. Moreover, depending on the ion pairing, TAN’s amine or catechol moieties can be exploited to impart antimicrobial activity while preserving compatibility with mammalian cells. TAN-based PEM coatings are shown to be compatible with mammalian cells. Still, they are effective at contact-killing both Gram-positive and Gram-negative bacteria and preventing their biofilm formation. The effectiveness against the biofilm-forming *P. aeruginosa* after 24 h of contact is particularly notable, as this bacterium forms pathogenic and obdurate biofilms on implant surfaces. TAN is a versatile biopolymer for the development of functional surface coatings on biomaterials.

## Figures and Tables

**Table 1:** The average size (mean ± standard deviation) and PDI of Tanfloc solution in different pH obtained from the number distributions.

pH	Hydrodynamic Diameter (nm)	PDI
5.0	4.52 ± 0.33	0.29
6.0	149.60 ± 19.27	0.29
7.4	2.49 ± 1.69	1.00
8.4	89.21 ± 25.60	0.41
9.3	338.24 ± 79.93	0.71

## Data Availability

A dataset containing the data generated in this study is publicly available from the Dryad database at https://doi.org/10.5061/10.5061/dryad.gqnk98sv9.

## Supplementary Materials

The following supporting information can be downloaded at: https://www.mdpi.com/article/10.3390/jfb14110554/s1, Figure S1: ^1^H NMR spectrum of TMC; Figure S2: Kinetics of 12-layer PEM assembly of (a) TAN-HA_12_, (b) TAN-CS_12_, and (c) PEI-TAN_12_ monitored by in situ FT-SPR. Arrows indicate the start of rinsing (orange), polycation deposition (green), and polyanion deposition (blue) steps; Figure S3: XPS survey of different PEM surfaces; Figure S4: Representative 2.0 μm × 2.0 μm AFM topographic images of the PEMs taken in PBS; Figure S5: Cytotoxicity results assessed through LDH assay on different PEM surfaces after 24 h of incubation. Values represent mean ± standard deviation (n = 4). **** *p* ≤ 0.0001, *** *p* ≤ 0.001, ** *p* ≤ 0.01, * *p* ≤ 0.05, and “ns” *p* ≥ 0.05 compared to controls; Figure S6: Bacterial growth in solution induced by different surfaces against (a) *S. aureus* and (b) *P. aeruginosa* after 6 h and 24 h of incubation in a bacterial solution. The experiments were performed at least twice using five samples of each surface. The optical density at 560 nm indicates the bacterial density in each solution, all of which are in the logarithmic growth phase. **** *p* ≤ 0.0001, *** *p* ≤ 0.001, ** *p* ≤ 0.01, * *p* ≤ 0.05, and “ns” *p* ≥ 0.05 compared to glass control at same time point; ++++ *p* ≤ 0.0001, +++ *p* ≤ 0.001, ++ *p* ≤ 0.01, + *p* ≤ 0.05, and “ns” *p* ≥ 0.05 compared to PS control at same time point; Figure S7: SEM images of the PEMs with different terminated layers incubated with *S. aureus* and *P. aeruginosa* after 6 h and 24 h at 37 °C. Original magnification was 5000×, and 1000×; Figure S8: Fluorescence microscopy images of *S. aureus* and *P. aeruginosa* on the PEMs. Live bacteria are represented in green (SYTO 9 stain) and dead bacteria in red (propidium iodide stain).
