# Supplementary material for: Expanding the Scope of an Amphoteric Condensed Tannin, Tanfloc, for Antibacterial Coatings
Source: J Funct Biomater. 2023 Nov 18;14(11):554. doi: 10.3390/jfb14110554 (PMC10672460; doi:10.3390/jfb14110554)
Supplement: Supplementary file 1 [file jfb-14-00554-s001.zip › jfb-2653679-supplementary.pdf]

Supporting information for:

Expanding the Scope of an Amphoteric Condensed Tannin, Tanfloc, for Antibacterial Coatings

Somayeh Baghersad [1], Liszt Y.C. Madruga [2], Allesandro F. Martins [3, 2], Ketul C. Popat *,[1,4,5], and Matt J. Kipper *,[1,2,5]

1 School of Biomedical Engineering, Colorado State University, Fort Collins, CO 80526

2 Department of Chemical and Biological Engineering, Colorado State University, Fort Collins, CO 80526

3 Department of Chemistry and Biotechnology, University of Wisconsin-River Falls, River Falls, WI 54022

4 Department of Mechanical Engineering, Colorado State University, Fort Collins, CO 80526

5 School of Materials Science and Engineering, Colorado State University, Fort Collins, CO 80526

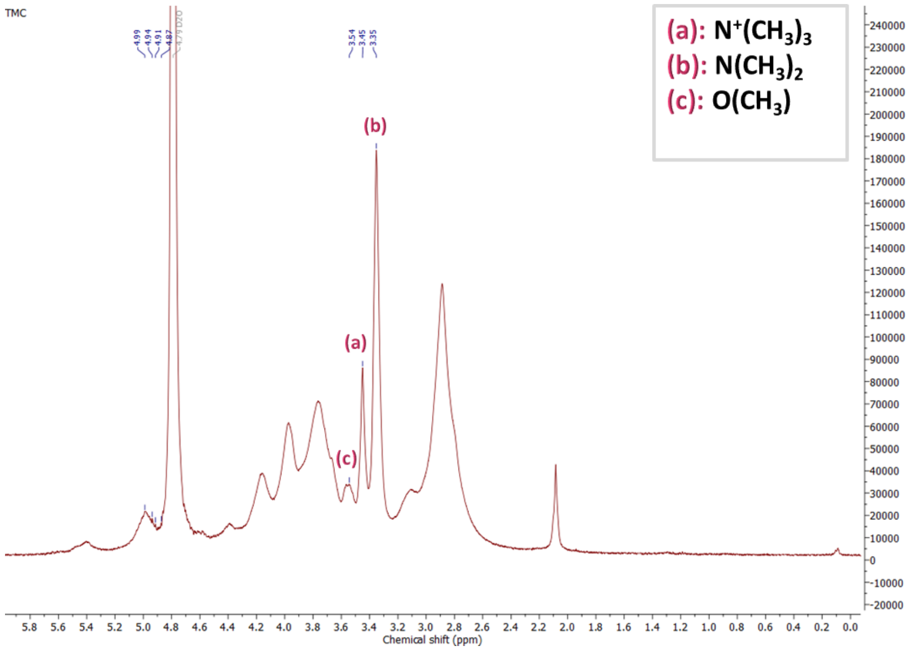

Figure S1. ^{1}H NMR spectrum of TMC.

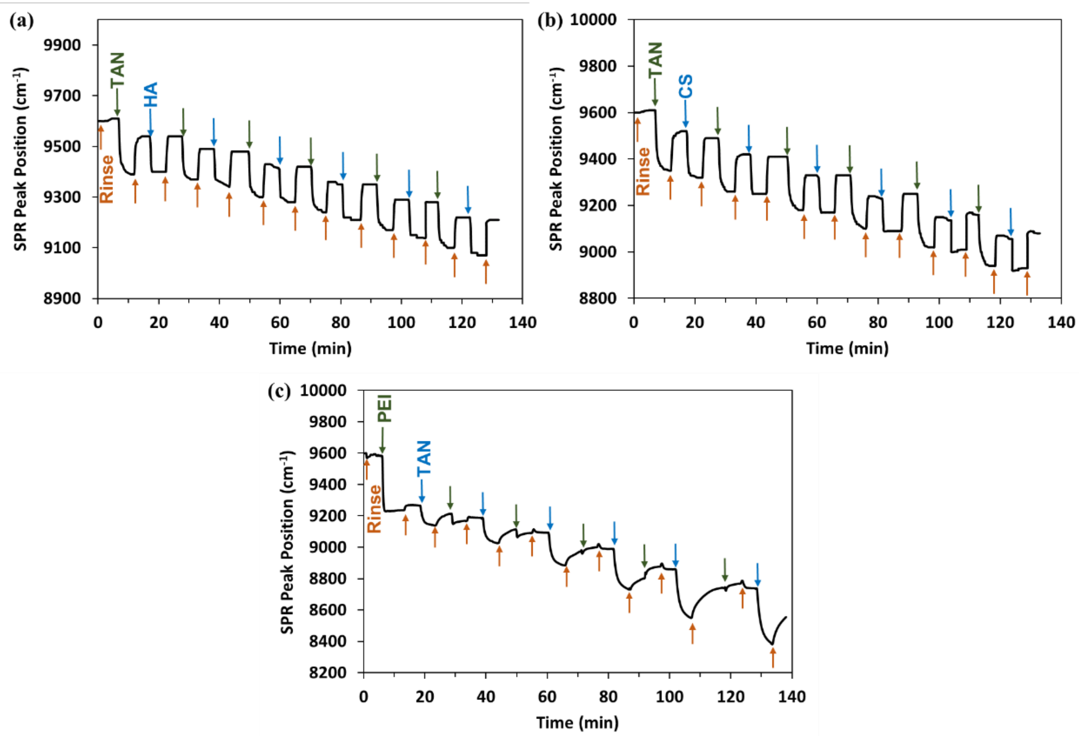

Figure S2. Kinetics of 12-layer PEM assembly of a) TAN-HA$_{12}$, b) TAN-CS$_{12}$, and c) PEI-TAN$_{12}$ monitored by in situ FT-SPR. Arrows indicate the start of rinsing (orange), polycation deposition (green), and polyanion deposition (blue) steps.

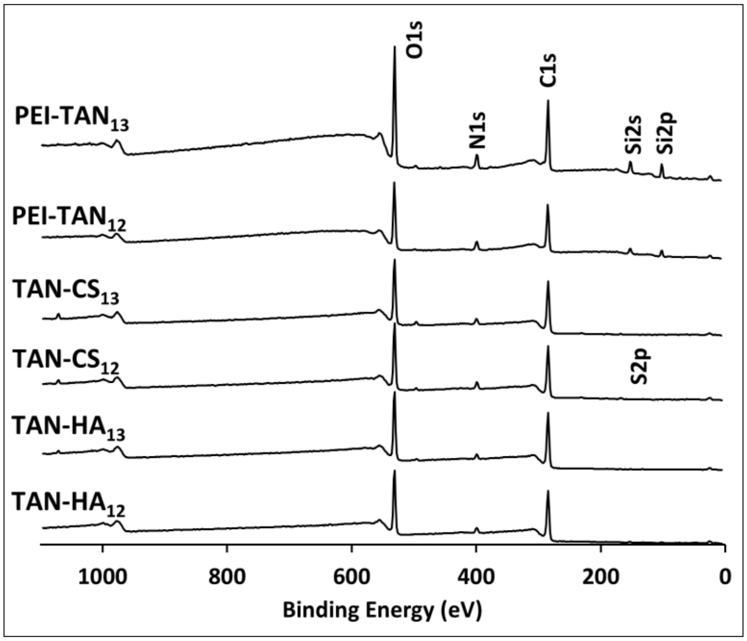

Figure S3. XPS survey of different PEM surfaces.

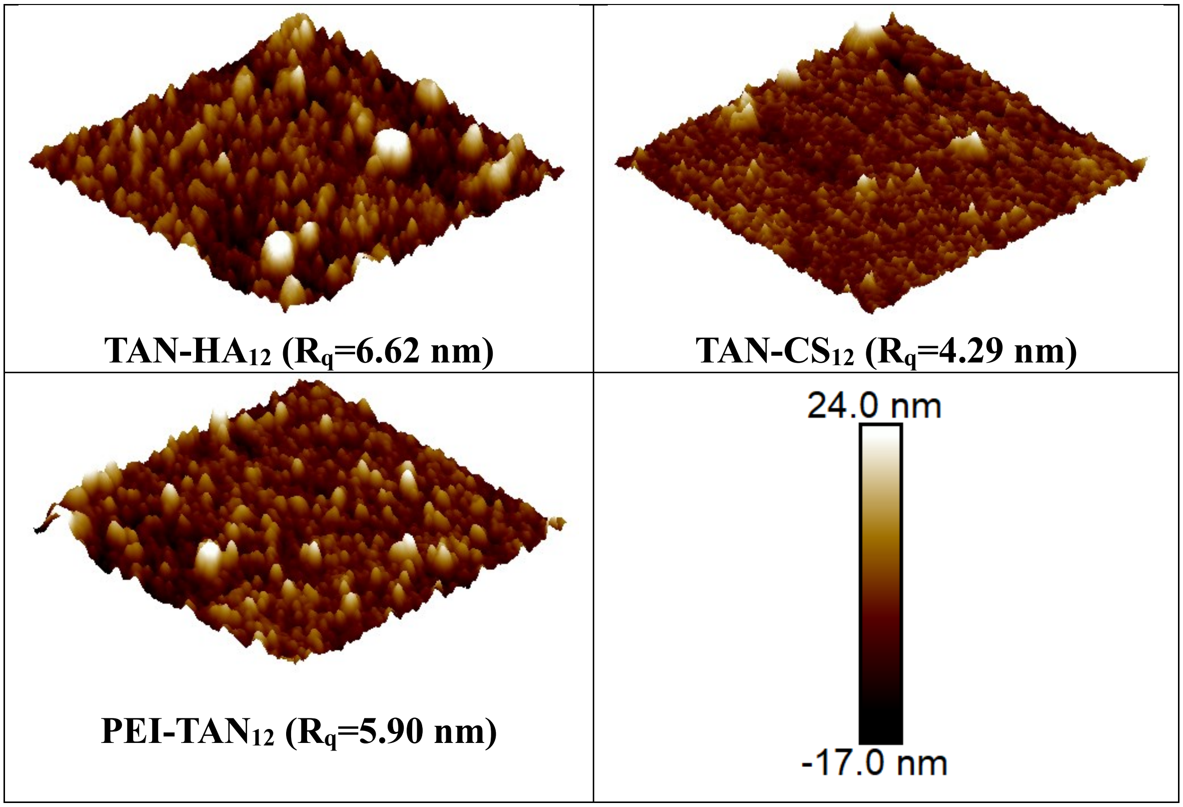

Figure S4. Representative 2.0 µm × 2.0 µm AFM topographic images of the PEMs taken in PBS.

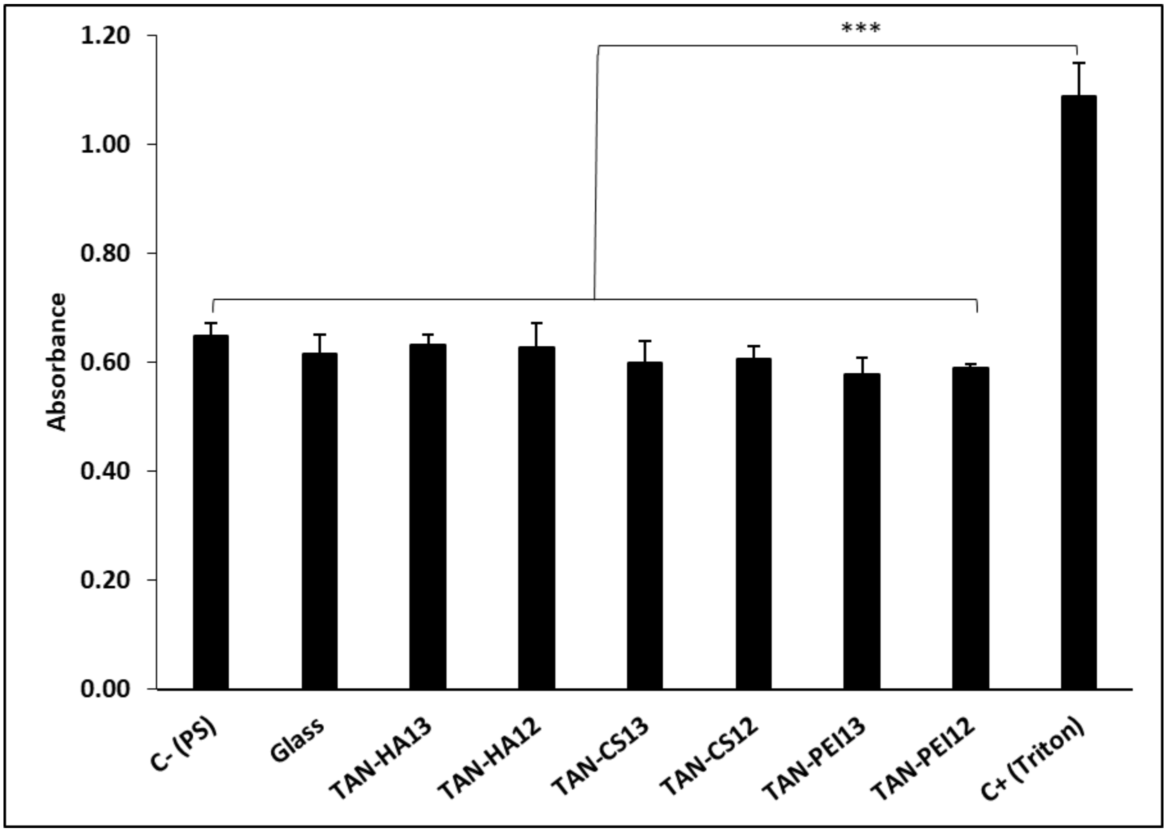

Figure S5. Cytotoxicity results assessed through LDH assay on different PEM surfaces after 24 h of incubation. Values represent mean ± standard deviation (n = 4). ****$p \leq 0.0001$, ***$p \leq 0.001$, **$p \leq 0.01$, *$p \leq 0.05$, and "ns" $p \geq 0.05$ compared to controls.

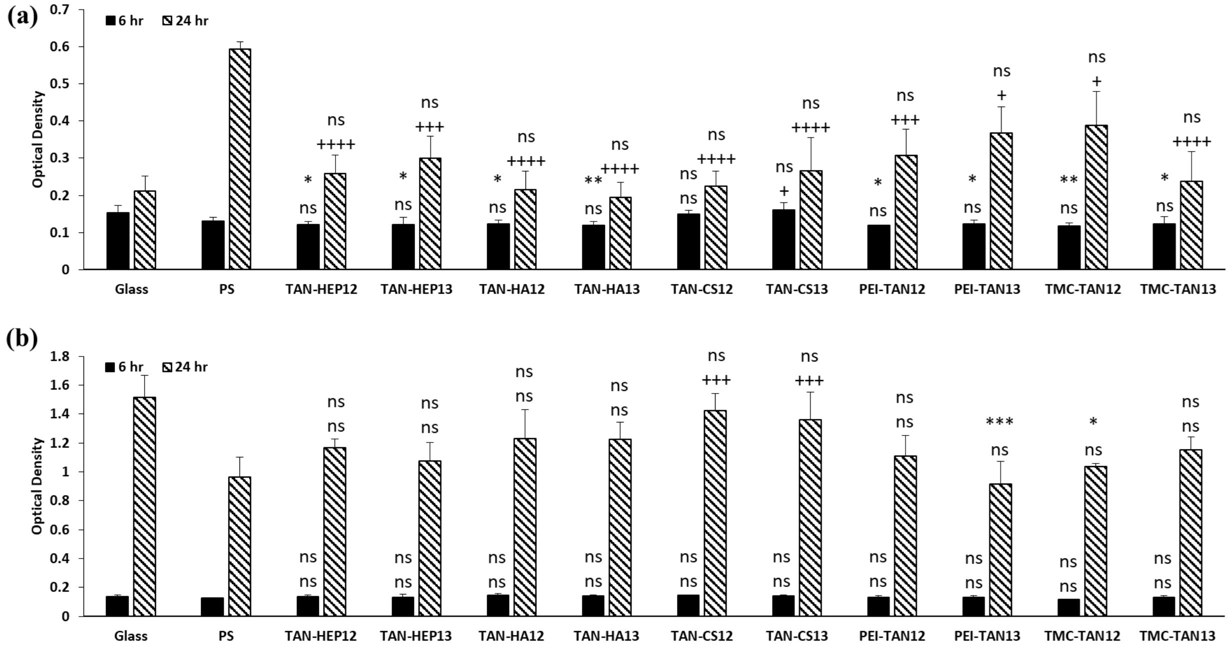

Figure S6. Bacterial growth in solution induced by different surfaces against (a) S. aureus and (b) P. aeruginosa after 6 and 24 hours of incubation in a bacterial solution. The experiments were performed at least twice using five samples of each surface. The optical density at 560 nm indicates the bacterial density in each solution, all of which are in the logarithmic growth phase. *******$p \leq 0.0001$, ******$p \leq 0.001$**, *****$p \leq 0.01$**, ****$p \leq 0.05$**, and "ns" $p \geq 0.05$ compared to glass control at same time point; ++++$p \leq 0.0001$, +++$p \leq 0.001$, ++$p \leq 0.01$, +$p \leq 0.05$, and "ns" $p \geq 0.05$ compared to PS control at same time point.**

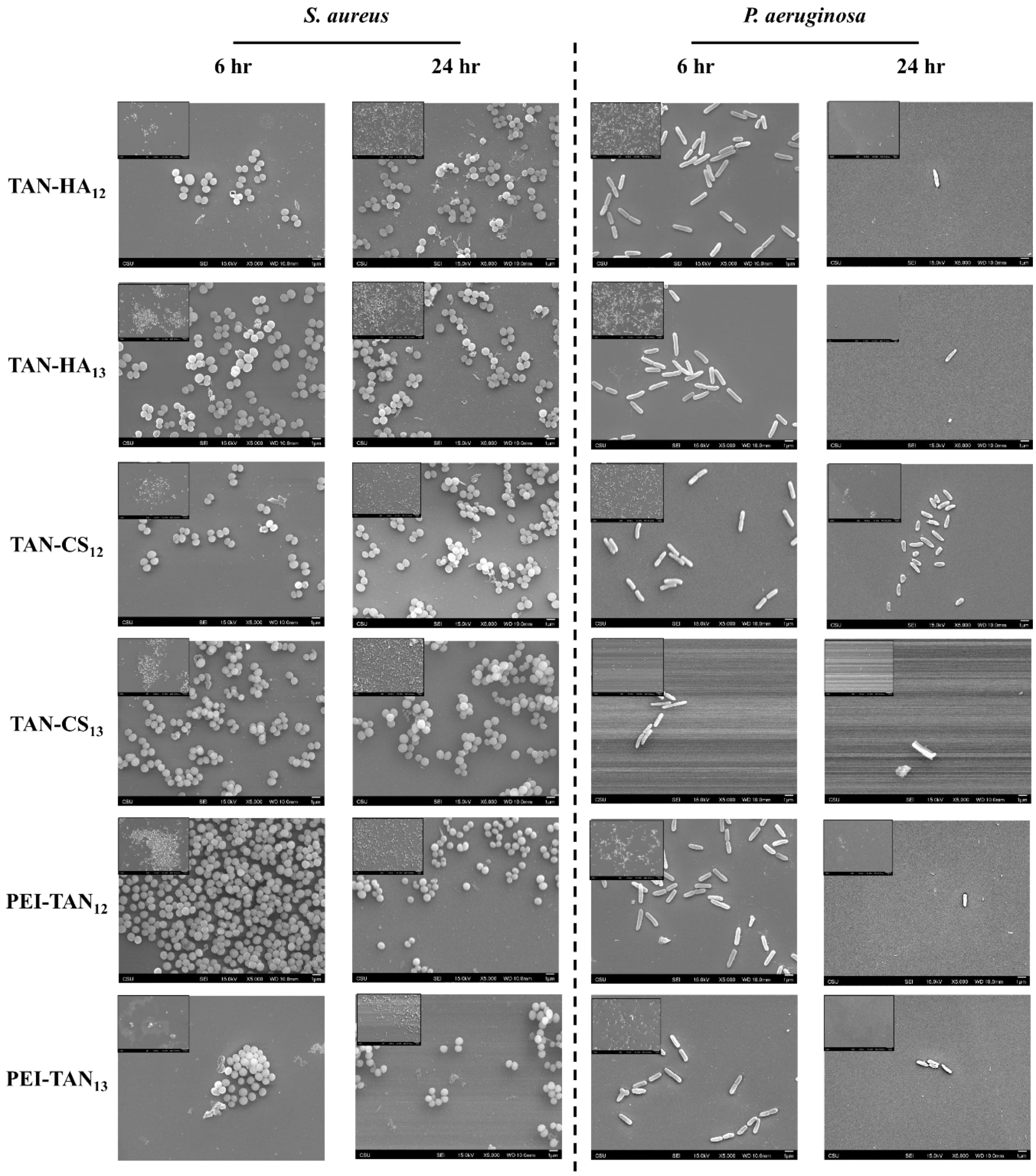

Figure S7. SEM images of the PEMs with different terminated layers incubated with *S. aureus* and *P. aeruginosa* after 6 and 24 h at 37 °C. Original magnification is 5,000x, and 1,000x

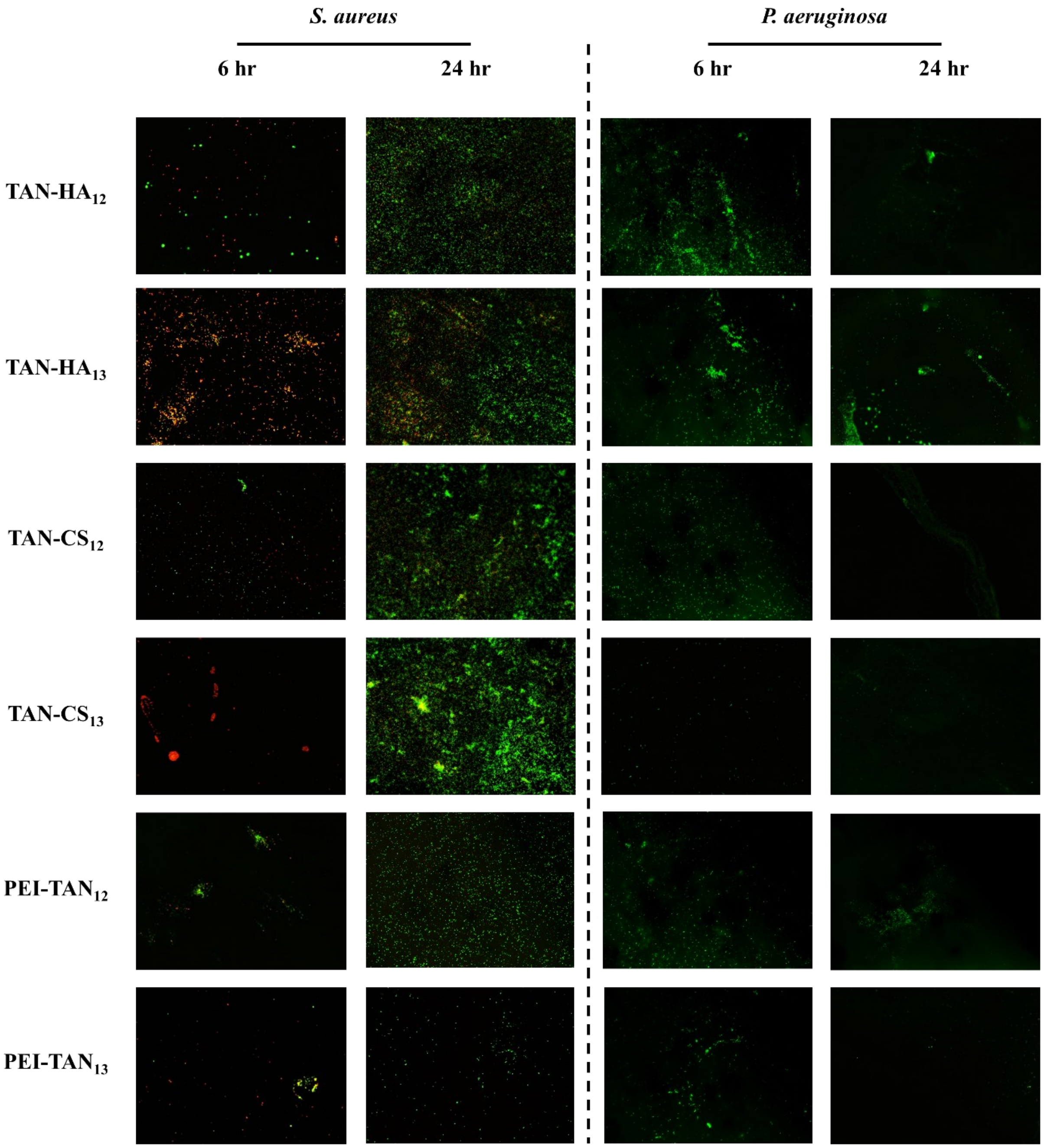

Figure S8. Fluorescence microscopy images of *S. aureus* and *P. aeruginosa* on the PEMs. Live bacteria are represented in green (SYTO 9 stain) and dead bacteria in red (propidium iodide stain).
